# Immediate Postpartum Long-Acting Reversible Contraceptive Use Following State-Specific Changes in Hospital Medicaid Reimbursement

**DOI:** 10.1001/jamanetworkopen.2022.37918

**Published:** 2022-10-21

**Authors:** Maria W. Steenland, Raj Vatsa, Lydia E. Pace, Jessica L. Cohen

**Affiliations:** 1 Population Studies and Training Center, Brown University, Providence, Rhode Island; 2 Interfaculty Initiative in Health Policy, Harvard University, Cambridge, Massachusetts; 3 Department of Medicine, Brigham and Women’s Hospital, Boston, Massachusetts; 4 Department of Global Health and Population, Harvard T.H. Chan School of Public Health, Boston, Massachusetts

## Abstract

**Importance:**

Facilitating access to the full range of contraceptive options is a health policy goal; however, inpatient provision of postpartum long-acting reversible contraceptive (LARC) methods has been limited due to lack of hospital reimbursement. Between March 2014 and January 2015, the Medicaid programs in 5 states began to reimburse hospitals for immediate postpartum LARC separately from the global maternity payment.

**Objective:**

To examine the association between Medicaid policies and provision of immediate postpartum LARC, and to examine hospital characteristics associated with policy adoption.

**Design, Setting, and Participants:**

This cross-sectional study used interrupted time series analysis. The setting was population-based in Georgia, Iowa, Maryland, New York, and Rhode Island. Participants included individuals who gave birth in these states between 2011 and 2017 (n = 3 097 188). Statistical analysis was performed from June 2021 to August 2022.

**Exposures:**

Childbirth after the start of Medicaid’s reimbursement policy.

**Main Outcomes and Measures:**

Immediate postpartum LARC (outcome), teaching hospital, Catholic-owned or operated, obstetrical care level, and urban or rural location (hospital characteristics).

**Results:**

The study included a total of 1 521 491 births paid for by Medicaid and 1 575 697 paid for by a commercial payer between 2011 and 2017. Prior to Medicaid reimbursement changes, 489 389 of 726 805 births (67%) were to individuals between 18 and 29 years of age, 219 363 of 715 905 births (31%) were to non-Hispanic Black individuals, 227 639 of 715 905 births (32%) were to non-Hispanic White individuals, 155 298 of 715 905 births (22%) were to Hispanic individuals, and 113 605 of 715 905 births (16%) were to individuals from other non-Hispanic racial groups. Among Medicaid-paid births, the policies were associated with an increase in the rate of immediate postpartum LARC provision in all states, although results for Maryland were not consistent across sensitivity analyses. The change in trend ranged from a quarterly increase of 0.05 percentage points in Maryland (95% CI, 0.01-0.08 percentage points) and 0.05 percentage points in Iowa (95% CI, 0.00-0.11 percentage points) to 0.82 percentage points (95% CI, 0.73-0.91 percentage points) in Rhode Island. The policy was also associated with an increase in immediate postpartum LARC provision among commercially paid births in 4 of 5 states. After the policy, only 38 of 366 hospitals (10%) provided more than 1% of birthing people with immediate postpartum LARC. These adopting hospitals were less likely to be Catholic (0% [0 of 31] vs 17% [41 of 245]), less likely to be rural (10% [3 of 31] vs 33% [81 of 247]), more likely to have the highest level of obstetric care (71% [22 of 31] vs 29% [65 of 223]) and be teaching hospitals (87% [27 of 31] vs 43% [106 of 246]) compared with nonadopting hospitals.

**Conclusions and Relevance:**

This cross-sectional study’s findings suggest that Medicaid policies that reimburse immediate postpartum LARC may increase access to this service; however, policy implementation has been uneven, resulting in unequal access.

## Key Points

**Question:**

Are Medicaid policies that reimburse hospitals for immediate postpartum long-acting reversible contraception (LARC) associated with increased provision of this service?

**Findings:**

In this cross-sectional study of more than 3 million births in Georgia, Iowa, Maryland, New York, and Rhode Island between 2011 and 2017, Medicaid reimbursement for immediate postpartum LARC was associated with an increase in immediate postpartum LARC provision among people with Medicaid in all states. The policies were also associated with increased provision among people with a commercially paid birth in 4 of 5 states.

**Meaning:**

These findings suggest that reimbursement of immediate postpartum LARC was associated with increased access to this service.

## Teaser

This cross-sectional study examines the association between Medicaid policies and provision of immediate postpartum long-acting reversible contraception (LARC) in addition to hospital characteristics associated with policy adoption.

## Introduction

Most pregnancies that begin within the first 6 months after childbirth are unintended.^1^ However, many postpartum people in the US cannot access their preferred method of contraception and achieve their reproductive goals.^2^ Contraceptive autonomy is a primary health policy goal, but many postpartum people are not offered the full range of contraceptive methods from which they can freely choose with sufficient and unbiased information. Contraceptive implants or intrauterine devices (IUDs) provided in the hospital after childbirth^3^ are referred to as immediate postpartum long-acting reversible contraception (LARC). Immediate postpartum LARC is an important option for postpartum people given well-documented gaps in postpartum care access and insurance coverage in the US, as well as the acceptability, safety, and effectiveness of LARC.^3^ Before 2012, immediate postpartum LARC was largely unavailable in inpatient settings.^4,5^ This limited postpartum people’s choice and has potentially contributed to the high rates of unintended and short-interval births in the US, both of which are associated with adverse maternal and newborn outcomes.^6^

Prior to 2012, Medicaid reimbursed hospitals for childbirth care using a fixed global payment, irrespective of LARC provision.^4^ As implants and IUDs can be costly,^7^ the lack of separate Medicaid reimbursement likely limited hospitals’ ability to offer these methods.^4^ As Medicaid pays for approximately 42% of childbirth hospitalizations annually, Medicaid hospital payment policies have important implications for maternal health nationally.^8^ Following state level advocacy^4^ and recommendations from professional groups,^3^ many state Medicaid programs began providing separate reimbursement for inpatient LARC in addition to the fixed global payment for delivery.^9^ Population-based studies from South Carolina, the first state to begin such reimbursement, have shown that the policy increased the availability of immediate postpartum LARC, reduced short birth intervals among adolescents, and reduced adverse infant outcomes.^10,11,12^ However, studies from other states, which have generally been limited to a subset of hospitals, provide evidence that hospitals have faced barriers to immediate postpartum LARC provision even after reimbursement policy changes. For example, after policy implementation, hospitals often received incomplete or delayed reimbursement of immediate postpartum LARC.^13,14,15^ Additionally, existing evidence indicates that immediate postpartum LARC provision remains limited to a small number of urban, academic medical centers.^16^ In light of these potential barriers to effective implementation, additional evidence is needed to understand whether Medicaid payments for immediate postpartum LARC have increased the availability and use of this contraceptive option across a range of states and hospital settings.

As of 2018, more than half of state Medicaid programs had begun reimbursement for immediate postpartum LARC.^9^ In this study, we examined whether Medicaid reimbursement policy changes for immediate postpartum LARC in 5 early-adopting states were associated with a change in immediate postpartum LARC provision. We hypothesized that Medicaid reimbursement for immediate postpartum LARC increased provision of this service, particularly among people with a Medicaid-paid childbirth. We also examined whether the policy was associated with a change in postpartum LARC provision among commercially insured individuals, and whether hospital level factors were associated with hospital policy implementation.

## Methods

### Data and Study Population

This cross-sectional study used data from State Inpatient Databases (SID) available through the Healthcare Cost and Utilization Project (HCUP), which included state inpatient data from 36 states. When we began this study, the most recent year of inpatient data available from HCUP was 2017. To obtain at least 2 years of follow-up data, we selected states for inclusion if their Medicaid programs implemented immediate postpartum LARC reimbursement in 2014 or 2015 and had inpatient data available from HCUP between 2011 and 2017.^5^ After implementing this inclusion criteria, our sample of states included Georgia, Iowa, Maryland, New York, and Rhode Island. Medicaid reimbursement policies went into effect in these states in April 2014 (Georgia), March 2014 (Iowa), September 2014 (Maryland), April 2014 (New York), and January 2015 (Rhode Island).^17^ The final multistate data set included inpatient discharge records for over 95% of hospital discharges during the study interval (2011-2017).

HCUP data comes from individual hospitals reported from individual states; therefore, collection methods for the race and ethnicity variables included in HCUP data may not be completely uniform. Race and ethnicity were classified as Hispanic, non-Hispanic Black (hereafter Black), non-Hispanic White (hereafter White), and other (Asian or Pacific Islander, Native American, as well as those classified by HCUP in their other category) or unknown race.

To obtain information about hospital characteristics, we linked HCUP hospital discharge records to the American Hospital Association’s (AHA) Annual Survey using linkage files provided by HCUP.^18^ This linkage enabled identification of facilities’ teaching status, rural status, Catholic affiliation, and obstetric level of care. In all states except Maryland (where zip code was unavailable), we linked the study data set with zip code–level income and education from the American Community Survey.

Using *International Classification of Diseases (ICD)* and diagnosis-related group (DRG) codes outlined in previous literature, we identified all hospitalizations for childbirth (eTable 1 in the Supplement).^19,20^ This study was considered not human participant research by the Harvard T.H. Chan School of Public Health and Brown University institutional review boards and as such did not require informed consent. This study followed the Strengthening the Reporting of Observational Studies in Epidemiology (STROBE) reporting guideline for cross-sectional studies.

### Outcomes

Our primary study outcome was uptake of immediate postpartum LARC. We adapted LARC billing codes from the Office of Population Affairs to create an indicator for receipt of an immediate postpartum implant or IUD during hospitalization for childbirth (eTable 2 in the Supplement).^21^

### Statistical Analysis

#### Main Analysis

We used an interrupted time series (ITS) design to estimate the extent to which the Medicaid policy change was associated with the trend and level of provision of immediate postpartum LARC.^22^ Analyses were conducted separately in each state at the hospital-quarter level by calculating the quarterly proportion of deliveries in each facility during which an immediate postpartum LARC was placed. ITS models included a linear time trend variable, a postpolicy indicator (to measure immediate changes in the outcome’s level following policy introduction), and an interaction between the postpolicy indicator and linear time trend (to measure changes in the outcome’s trend following policy introduction). We adjusted for seasonality with quarter-of-year fixed effects and for hospital characteristics that did not change over the study period using hospital fixed effects (eAppendix in the Supplement).

In addition to examining the immediate association between the policy and the change in the outcome’s level and trend, we also examined the net change in the study outcome during the last quarter of the study period (October to December 2017) by taking a linear combination of the regression coefficients for the change in level (postpolicy indicator) and trend (interaction term). We calculated Driscoll-Kraay standard errors to account for autocorrelation, heteroskedasticity, and cross-sectional dependence of observations within states, and we used 2-tailed hypothesis tests with a significance level of 2-sided *P* < .05.^23^ Statistical analysis was performed using R version 4.1.2 (R Project for Statistical Computing).

#### Spillovers to Commercially Paid Births and Analysis of Hospital Determinants of Policy Uptake

Our main analysis was restricted to Medicaid-paid births. However, it is possible that changes to Medicaid reimbursement for immediate postpartum LARC could have affected commercially paid births (eg, through adoption of new protocols, clinician training, device availability and patient and clinician awareness). To examine whether Medicaid LARC reimbursement was associated with immediate postpartum LARC in the commercially insured population, we conducted the same regression analyses described previously among commercially paid births.

Finally, to examine facility determinants and concentration of immediate postpartum LARC provision, we calculated the share of deliveries with an associated immediate postpartum LARC in each hospital in the months following reimbursement policy change. Due to the high upfront costs for hospitals to purchase LARCs, accommodate LARC billing, and train clinicians on LARC placement, we classified hospitals with greater than 1% of deliveries with an associated immediate postpartum LARC as adopting facilities, while the remainder were classified as nonadopting facilities. Any smaller number of LARCs billed likely indicated billing errors rather than a change in LARC availability at the hospital.

#### Sensitivity Analyses

We conducted several sensitivity analyses to assess the robustness of our results. First, we conducted a series of falsification tests within each state to test whether structural breaks in the time series occurred around the time of policy change.^24^ Second, to assess robustness to adjustments for quarterly changes in the population giving birth over the study period, we included demographic control variables in the analysis. Third, as Medicaid program guidelines in Iowa^25^ and New York^26^ specified that hospitals should bill immediate postpartum LARCs on separate ambulatory claims, we cross-checked discharge records within 7 days of childbirth from HCUP’s State Ambulatory Surgery and Services Databases (SASD) in these 2 states to supplement our identification of immediate postpartum LARC.^27^ Fourth, we estimated effects at the state-month level (rather than state-quarter level). Finally, we conducted the analysis without observations from low-volume hospitals, defined as hospitals with an average number of deliveries per year less than 500.^28^ The purpose of this final analysis was to test whether low-volume hospitals with high variance in the study outcome were driving the study results.

## Results

The study sample included a total of 1 521 491 births paid for by Medicaid, with 439 655 births from Georgia, 90 624 births from Iowa, 194 057 births from Maryland, 762 821 births from New York, and 34 334 births from Rhode Island. In addition, there were a total of 1 575 697 births paid for by a commercial payer in the 5 study states between 2011 and 2017 (eTable 4 in the Supplement). Prior to Medicaid reimbursement changes, 489 389 of 726 805 births (67%) were to individuals aged between 18 and 29 years of age, 227 639 of 715 905 births (32%) were to White individuals, 219 363 of 715 905 births (31%) were to Black individuals, 155 298 of 715 905 births (22%) were to Hispanic individuals, and 113 605 of 715 905 births (16%) were to individuals from other non-Hispanic racial groups (Table 1). Approximately 77% (433 276 of 566 733) of persons with a Medicaid-paid birth lived in a zip code in which the average educational level was below the national median, and approximately 28% (188 723 of 669 375) lived in a zip code in which average income was below the first income quartile nationally (Table 1). Characteristics of Medicaid-insured individuals are provided by state in eTable 3 in the Supplement.

**Table 1.** Demographic Information of All Medicaid Insured Individuals With Delivery Episodes Prior to Reimbursement Policy Change for Inpatient Long-Acting Reversible Contraception

Characteristic^a^	Deliveries, No./No. (%) (n = 726 805)
Age, y	
12-17	22 976/726 805 (3.2)
18-29	489 389/726 805 (67.3)
30-35	153 009/726 805 (21.1)
36-50	61 431/726 805 (8.5)
Race and ethnicity	
Hispanic	155 298/715 905 (21.7)
Non-Hispanic	
Black	219 363/715 905 (30.6)
White	227 639/715 905 (31.8)
Other^b^	113 605/715 905 (15.9)
Zip code below median education^c^	433 276/566 733 (76.5)
Zip code below first quartile income^c^	188 723/669 375 (28.2)

^a^ Demographic information pooled across all states in the sample (Georgia, Iowa, Maryland, New York, Rhode Island).

^b^ The other race and ethnicity category included Asian or Pacific Islander, Native American, as well as those classified by the Healthcare Cost and Utilization Project in their other category.

^c^ Zip code level educational attainment data unavailable for Maryland; cut points determined based on zip code level data from zip codes across the US.

**Figure 1.**
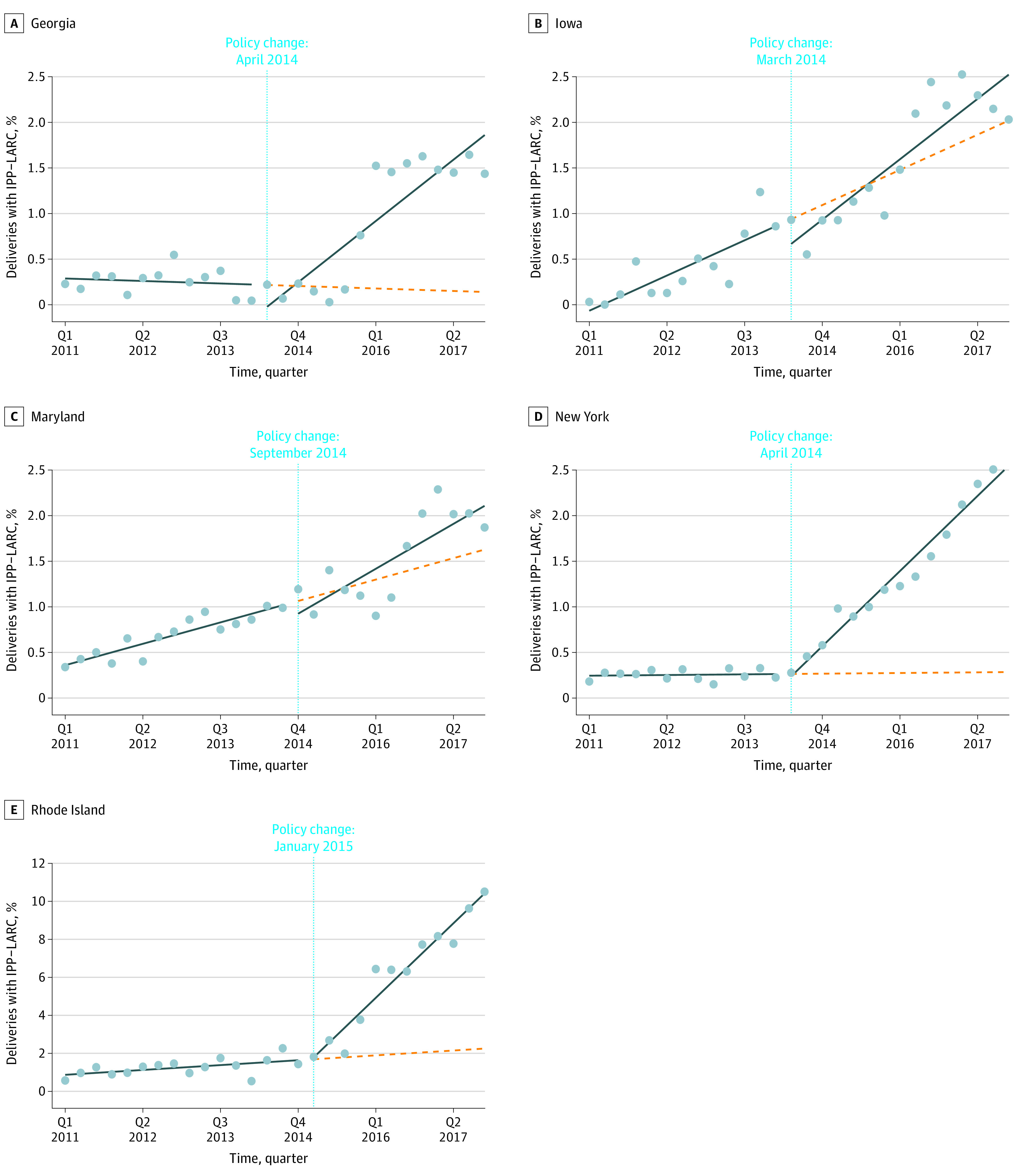
Quarterly Percentage of Deliveries With Immediate Postpartum Long-Acting Reversible Contraception (IPP-LARC) Placement Prepolicy and Postpolicy Implementation Among Medicaid-Insured Individuals This figure displays the percentage of seasonality-adjusted deliveries among Medicaid-insured individuals in each state-quarter with an associated inpatient IPP-LARC placement (dots). In each state panel, the horizontal line denotes the time of Medicaid reimbursement policy change, before and after which separate linear trend lines (solid lines) are estimated to capture the quarterly change in share of deliveries with inpatient IPP-LARC placement over time. The dashed lines represent counterfactual postpolicy linear trend lines in each state, which are extensions of the prepolicy linear trend lines through the end of the study period. The total number of Medicaid-covered deliveries across the study period—from quarter (Q) 1 2011 through the end of Q4 2017—for each state is as follows: 439 655 (Georgia), 90 624 (Iowa), 194 057 (Maryland), 762 821 (New York), and 34 334 (Rhode Island).

Prepolicy and postpolicy trends in the percentage of childbirth hospitalizations paid for by Medicaid with an immediate postpartum LARC between 2011 and 2017 are presented in Figure 1, and ITS regression results are presented in Table 2. Among Medicaid paid births, very few immediate postpartum LARCs were provided before Medicaid reimbursement (eTable 3 in the Supplement). The prepolicy quarterly trend in immediate postpartum LARC provision was not statistically significant in Georgia (−0.01 [95% CI, −0.03 to 0.01]), New York (0.00 [95% CI, 0.00 to 0.01]), or Rhode Island (0.03 [95% CI, −0.01 to 0.07]), but was increasing in Iowa (0.08 [95% CI, 0.05 to 0.11]) and Maryland (0.06 [95% CI, 0.05 to 0.07]) (Table 2).

**Table 2.** Interrupted Time Series Estimates of Immediate Postpartum Long-Acting Reversible Contraception Uptake Among Medicaid-Insured Individuals Before and After Medicaid Reimbursement Change for Long-Acting Reversible Contraception Provision^a^

Variable	Georgia^b^	*P* value	Iowa^b^	*P* value	Maryland^b^	*P* value	New York^b^	*P* value	Rhode Island^b^	*P* value
Quarterly change during prepolicy period, percentage points	−0.01 (−0.03 to 0.01)	.22	0.08 (0.05 to 0.11)	<.001	0.06 (0.05 to 0.07)	<.001	0.00 (0.00 to 0.01)	.22	0.03 (−0.01 to 0.07)	.11
Level change at time of policy onset, percentage points	−0.14 (−0.47 to 0.19)	.40	−0.28 (−0.62 to 0.07)	.11	−0.17 (−0.48 to 0.14)	.27	−0.06 (−0.19 to 0.08)	.43	0.13 (−0.79 to 1.04)	.79
Quarterly change during postpolicy period relative to prepolicy period, percentage points	0.14 (0.11 to 0.18)	<.001	0.05 (0.00 to 0.11)	.05	0.05 (0.01 to 0.08)	.01	0.17 (0.15 to 0.18)	<.001	0.82 (0.73 to 0.91)	<.001
Overall change at end of Q4 2017 relative to estimated counterfactual, percentage points^c^	1.88 (1.36 to 2.39)	<.001	0.48 (−0.39 to 1.34)	.28	0.38 (0.09 to 0.67)	.01	2.27 (2.09 to 2.44)	<.001	9.15 (8.36 to 9.94)	<.001

Abbreviation: Q, quarter.

^a^ Reimbursement policy change implemented in Georgia in April 2014, Iowa in March 2014, Maryland in September 2014, New York in April 2014, and Rhode Island in January 2015.

^b^ All estimates are adjusted for seasonality with quarter fixed effects, and for clustering of observations by hospital with hospital fixed effects. Driscoll-Kraay standard errors are used to account for autocorrelation, heteroskedasticity, and cross-sectional dependence of observations within states.

^c^ Absolute change in fraction of Medicaid-insured deliveries with immediate postpartum long-acting reversible contraception placement. Estimated effect sizes are relative to the estimated counterfactual fraction of deliveries with associated immediate postpartum long-acting reversible contraception through the end of Q4 2017 in all states.

Among Medicaid paid births, the quarterly trend in immediate postpartum LARC provision increased after Medicaid reimbursement for the service by 0.14 percentage points (95% CI, 0.11-0.18 percentage points) in Georgia, 0.05 percentage points (95% CI, 0.00-0.11 percentage points) in Iowa, 0.05 percentage points (95% CI, 0.01-0.08 percentage points) in Maryland, 0.17 percentage points (95% CI, 0.15-0.18 percentage points) in New York, and 0.82 percentage points (95% CI, 0.73-0.91 percentage points) in Rhode Island (Table 2). In the final quarter of the study period, the policy was associated with increases of 1.88 percentage points (95% CI, 1.36-2.39 percentage points) in Georgia, 0.38 percentage points (95% CI, 0.09-0.67 percentage points) in Maryland, 2.27 percentage points (95% CI, 2.09-2.44 percentage points) in New York, and 9.15 percentage points (95% CI, 8.36-9.94 percentage points) in Rhode Island (Table 2). The policy was not associated with a statistically significant overall change in immediate postpartum LARC in the last quarter of the study period in Iowa.

Table 3 presents ITS regression results for immediate postpartum LARC provision among commercially paid births. In the commercial population, after the policy change the quarterly trend in immediate postpartum LARC increased by 0.02 percentage points (95% CI, 0.01-0.02 percentage points) in Georgia, 0.03 percentage points (95% CI, 0.02-0.04 percentage points) in Maryland, 0.03 percentage points (95% CI, 0.02-0.03 percentage points) in New York, and 0.09 percentage points (95% CI, 0.07-0.11 percentage points) in Rhode Island (Table 3). For commercially paid births, there were small but statistically significant decreases in the level of immediate postpartum LARC at the time of the policy change in Maryland and Rhode Island (Table 3). In the final quarter of the study period, the policy was associated with increases in immediate postpartum LARC of 0.20 percentage points (95% CI, 0.17-0.23) in Georgia, 0.19 percentage points (95% CI, 0.06, 0.33 percentage points) in Maryland, 0.38 percentage points (95% CI, 0.34-0.42 percentage points) in New York, and 0.82 percentage points (95% CI, 0.54-1.10) in Rhode Island (Table 3).

**Table 3.** Interrupted Time Series Estimates of Immediate Postpartum Long-Acting Reversible Contraception Uptake Among Commercially Insured Individuals Before and After Medicaid Reimbursement Change for Long-Acting Reversible Contraception Provision in States With a Policy Effect Among Medicaid-Insured Individuals^a^

Variable	Georgia^b^	*P* value	Iowa^b^	*P* value	Maryland^b^	*P* value	New York^b^	*P* value	Rhode Island^b^	*P* value
Quarterly change during prepolicy period, percentage points	0.00 (0.00 to 0.00)	.05	0.02 (0.01 to 0.03)	<.001	0.02 (0.02 to 0.03)	<.001	0.00 (0.00 to 0.00)	.01	0.02 (0.00 to 0.03)	.01
Level change at time of policy onset, percentage points	−0.02 (−0.04 to 0.01)	.18	0.03 (−0.09 to 0.15)	.59	−0.15 (−0.24 to −0.06)	<.001	0.01 (−0.01 to 0.03)	.35	−0.19 (−0.35 to −0.02)	.02
Quarterly change during postpolicy period relative to prepolicy period, percentage points	0.02 (0.01 to 0.02)	<.001	0.00 (−0.02 to 0.01)	.73	0.03 (0.02 to 0.04)	<.001	0.03 (0.02 to 0.03)	<.001	0.09 (0.07 to 0.11)	<.001
Overall change at end of Q4 2017 relative to estimated counterfactual, percentage points^c^	0.20 (0.17 to 0.23)	<.001	−0.01 (−0.30 to 0.28)	.95	0.19 (0.06 to 0.33)	.004	0.38 (0.34 to 0.42)	<.001	0.82 (0.54 to 1.10)	<.001

Abbreviation: Q, quarter.

^a^ Reimbursement policy change implemented in Georgia in April 2014, Maryland in September 2014, New York in April 2014, and Rhode Island in January 2015.

^b^ All estimates are adjusted for seasonality with quarter fixed effects, and for clustering of observations by hospital with hospital fixed effects. Driscoll-Kraay standard errors are used to account for autocorrelation, heteroskedasticity, and cross-sectional dependence of observations within states.

^c^ Absolute change in fraction of commercially insured deliveries with immediate postpartum long-acting reversible contraception placement. Estimated effect sizes are relative to the estimated counterfactual fraction of deliveries with associated immediate postpartum long-acting reversible contraception through the end of Q4 2017 in all states.

**Figure 2.**
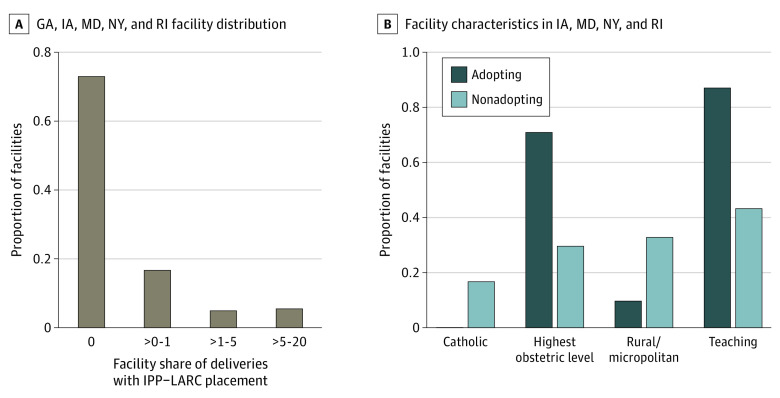
Distribution of Immediate Postpartum Long-Acting Reversible Contraception (IPP-LARC) Uptake Across Facilities and Evaluation of Adopting Facility Characteristics This figure focuses on Medicaid-insured deliveries in the postpolicy period (March 2014 onwards in IA; April 2014 onwards in GA and NY; September 2014 onwards in MD; January 2015 onwards in RI). A, Displays the distribution of facilities with respect to the share of facility deliveries with inpatient IPP-LARC placement (n = 366 facilities). B, Assesses the fraction of adopting vs nonadopting facilities with various characteristics (n = 278 delivery adopting facilities). An adopting facility was defined as one in which more than 1% of Medicaid-insured deliveries in the postpolicy period were associated with an IPP-LARC placement. Only IA, MD, NY, and RI were included because AHA Annual Survey linkage filed were unavailable for GA.

Across all states in our sample, 267 of 366 hospitals (73%) did not provide any immediate postpartum LARC after their respective state’s policy change (Figure 2). Immediate postpartum LARC provision ranged from 0% to 1% of deliveries in 61 of 366 hospitals (17%), from 1% to 5% of deliveries in 18 of 366 hospitals (5%), and from 5% to 20% in 20 of 366 hospitals (5%). In total, 10% of hospitals (38 of 366) provided immediate postpartum LARC in more than 1% of their childbirths following the policy change (adopting hospitals), whereas 90% (328 of 366) provided immediate postpartum LARC in fewer than 1% of childbirths (nonadopting hospitals) (Figure 2). Compared with nonadopting hospitals, adopting hospitals were less likely to be Catholic (0% [0 of 31] vs 17% [41 of 245]) and less likely to be rural (10% [3 of 31] vs 33% [81 of 247]). Adopting hospitals were also more likely to have the highest level of obstetric care (71% [22 of 31] vs 29% [65 of 223]) and be teaching hospitals (87% [27 of 31] vs 43% [106 of 246]) compared with nonadopting hospitals (Figure 2).

We found statistically significant trend breaks around the onset of the policy change in Georgia, New York, and Rhode Island. In Iowa and Maryland, we found no statistically significant structural break at the time of policy onset, but there were statistically significant structural breaks identified well after the policy was introduced in each state (eTable 5 in the Supplement). Finally, our results were largely robust to (1) the inclusion of controls accounting for changes in the population giving birth in each state; (2) supplementing our identification of immediate postpartum LARC with SASD discharge records in Iowa and New York; (3) aggregating deliveries at the monthly vs quarterly level; and (4) dropping observations from low-volume facilities (eTables 6-9 in the Supplement). Of note, in the analysis that added controls and the analysis that dropped low-volume facilities, the policy was no longer associated with a change in the trend or an overall change in immediate postpartum LARC in Maryland.

## Discussion

In all 5 early-adopting states included in the study, Medicaid reimbursement policy changes were associated with an increase in the rate of immediate postpartum LARC provision among Medicaid paid childbirths. However, we found mixed results in Maryland, where we found statistically significant estimates in unadjusted regression models but not in models with controls for the population giving birth in each quarter or models without observations from low-volume facilities. Medicaid reimbursement was associated with an increase in provision of immediate postpartum LARC among people with a commercially paid childbirth in 4 of the 5 study states, although the effect sizes in this population were much more modest than in the Medicaid-insured population. We found that provision of immediate postpartum LARC was concentrated among a small subset of hospitals, and that most hospitals provided no immediate postpartum LARC after the start of Medicaid reimbursement. Hospitals that provided immediate postpartum LARC after the policy change were disproportionately urban, teaching hospitals equipped to provide the highest level of obstetric care, and were less likely to be owned by or affiliated with a Catholic organization.

These results add to the limited evidence base examining the relationship between Medicaid reimbursement of immediate postpartum LARC and states’ provision of the service. Previous studies found that Medicaid reimbursement in South Carolina was associated with a large increase in immediate postpartum LARC, while other studies focused on Iowa, Louisiana, and Wisconsin found more modest gains in provision after the policy change.^11,12,16,29^ Our findings also show a range of effect sizes between states with overall increases in the percentage of individuals receiving an immediate postpartum LARC as of the last quarter of the study period as small as 0.38 percentage points (95% CI, 0.09-0.67 percentage points) in Maryland and as large as 9.15 percentage points (95% CI, 8.36-9.94 percentage points) in Rhode Island.

Our finding of limited dispersion of the policy across state hospitals is also consistent with studies examining immediate postpartum LARC in other states. Only a subset of hospitals began providing immediate postpartum LARC after Medicaid reimbursement in South Carolina and New Mexico,^12,15^ and most immediate postpartum LARC provision took place in urban academic teaching hospitals.^16,29^ Previous studies have found that reimbursement for immediate postpartum LARC is often delayed or incomplete.^13,14,15,30^ Lower-resourced hospitals may be reluctant to take on the high upfront cost of LARC purchase without guaranteed timely reimbursement. Previous studies have also pointed out that individual champions who help secure resources, and overcome institutional obstacles, are needed to support policy implementation.^16^

Hospital-level variability in access to immediate postpartum LARC in states that have adopted Medicaid reimbursement may result in within-state inequities in access. This is particularly true for people served by rural relative to urban hospitals, and people served by Catholic-owned hospitals. Catholic hospital ownership has been increasing rapidly over the last 2 decades,^31^ raising concerns about patient access to long-active reversible contraception in these facilities.^32^ Lower levels of immediate postpartum LARC implementation in Catholic hospitals is consistent with previous evidence that Catholic owned or affiliated clinics are less likely to offer copper IUDs.^33^

Interestingly, we found that after the policy change, provision of immediate postpartum LARC also increased among people whose childbirth care was paid for by a commercial payer. Although we cannot determine the cause of these increases using our data source, it is possible that the necessary hospital preparation to support LARC provision to Medicaid-covered individuals made it easier for hospitals to provide LARC to commercially covered patients, even without reimbursement. It is also possible that some commercial payers began to offer reimbursement at the same time as Medicaid’s policy change.

Increased access to immediate postpartum LARC can expand available postpartum contraceptive options and improve postpartum contraceptive convenience and effectiveness. Immediate postpartum LARC is recommended by ACOG as an important option for birthing people.^3^ However, increased provision of immediate postpartum LARC has the potential to reduce contraceptive autonomy if contraceptive care is not patient-centered. Previous qualitative studies have found that some respondents who received an immediate postpartum LARC felt pressured to use a LARC, or had not been given enough information to make a fully informed decision.^34,35^ Other studies have found that clinicians can be reluctant to remove a LARC before its expiration.^36,37^ Shared decision-making and efforts to promote contraceptive autonomy should be core components of implementation of immediate postpartum LARC.

### Limitations

This study has several limitations. First, it is possible that HCUP SID data do not fully capture immediate postpartum LARC before Medicaid reimbursement, because discharge records are primarily based on hospital billing records. It is also possible that some immediate postpartum LARC provision after the policy change was not captured in HCUP (eg, if an inpatient LARC was only billed on a professional claim without a record of placement on the hospital claim). An additional limitation is that this study does not directly assess contraceptive choice, and therefore, does not assess whether Medicaid reimbursement policy changes increased contraceptive autonomy. Furthermore, as the data do not consistently allow for individual linkage over time, this study does not examine the effect of the policies on birth intervals.

## Conclusions

Medicaid reimbursement of immediate postpartum LARC can increase availability of this service, but changes to reimbursement policy alone are not enough to ensure that all hospitals begin to provide the service. Additional training and financial support may be needed, particularly for smaller rural hospitals to be able to provide this service.

## Notes

Supplement. **eAppendix.** Primary Regression Specification **eTable 1.** Diagnosis and Procedure Codes for Identification of Hospitalizations for Childbirth **eTable 2.** Diagnosis and Procedure Codes for Identification of Immediate Postpartum LARC **eTable 3. **Demographic Information by State of all Medicaid-Insured Individuals With Delivery Episodes Prior to Medicaid Reimbursement Policy Change **eTable 4.** Demographic Information for All Commercially Insured Individuals With Delivery Episodes Prior to Reimbursement Policy Change for Inpatient LARC **eTable 5.** Testing for Structural Break Points in the Share of Medicaid-Insured Deliveries With Immediate Postpartum LARC Uptake **eTable 6.** Adjusted Interrupted Time Series Estimates of Immediate Postpartum LARC Uptake Among Medicaid Insured Individuals Before and After Medicaid Reimbursement Change for LARC Provision **eTable 7.** Interrupted Time Series Estimates for Iowa and New York Following Inclusion of Immediate Postpartum LARCs from State Ambulatory Data **eTable 8.** Aggregated at the Monthly Level **eTable 9.** Interrupted Time Series Estimates for Medicaid Insured Individuals After Dropping Low-Volume Facilities
