# Supplementary material for: Immediate Postpartum Long-Acting Reversible Contraceptive Use Following State-Specific Changes in Hospital Medicaid Reimbursement
Source: JAMA Netw Open. 2022 Oct 21;5(10):e2237918. doi: 10.1001/jamanetworkopen.2022.37918 (PMC9587474; doi:10.1001/jamanetworkopen.2022.37918)
Supplement: Supplement. — eAppendix. Primary Regression Specification eTable 1. Diagnosis and Procedure Codes for Identification of Hospitalizations for Childbirth eTable 2. Diagnosis and Procedure Codes for Identification of Immediate Postpartum LARC eTable 3. Demographic Information by State of all Medicaid-Insured Individuals With Delivery Episodes Prior to Medicaid Reimbursement Policy Change eTable 4. Demographic Information for All Commercially Insured Individuals With Delivery Episodes Prior to Reimbursement Policy Change for Inpatient LARC eTable 5. Testing for Structural Break Points in the Share of Medicaid-Insured Deliveries With Immediate Postpartum LARC Uptake eTable 6. Adjusted Interrupted Time Series Estimates of Immediate Postpartum LARC Uptake Among Medicaid Insured Individuals Before and After Medicaid Reimbursement Change for LARC Provision eTable 7. Interrupted Time Series Estimates for Iowa and New York Following Inclusion of Immediate Postpartum LARCs from State Ambulatory Data eTable 8. Aggregated at the Monthly Level eTable 9. Interrupted Time Series Estimates for Medicaid Insured Individuals After Dropping Low-Volume Facilities [file jamanetwopen-e2237918-s001.pdf]

Supplemental Online Content

Steenland MW, Vatsa R, Pace LE, Cohen JL. Immediate postpartum long-acting reversible contraceptive use following state-specific changes in hospital Medicaid reimbursement. *JAMA Netw Open*. 2022;5(10):e2237918. doi:10.1001/jamanetworkopen.2022.37918

eAppendix. Primary Regression Specification

eTable 1. Diagnosis and Procedure Codes for Identification of Hospitalizations for Childbirth

eTable 2. Diagnosis and Procedure Codes for Identification of Immediate Postpartum LARC

eTable 3. Demographic Information by State of all Medicaid-Insured Individuals With Delivery Episodes Prior to Medicaid Reimbursement Policy Change

eTable 4. Demographic Information for All Commercially Insured Individuals With Delivery Episodes Prior to Reimbursement Policy Change for Inpatient LARC

eTable 5. Testing for Structural Break Points in the Share of Medicaid-Insured Deliveries With Immediate Postpartum LARC Uptake

eTable 6. Adjusted Interrupted Time Series Estimates of Immediate Postpartum LARC Uptake Among Medicaid Insured Individuals Before and After Medicaid Reimbursement Change for LARC Provision

eTable 7. Interrupted Time Series Estimates for Iowa and New York Following Inclusion of Immediate Postpartum LARCs from State Ambulatory Data

eTable 8. Aggregated at the Monthly Level

eTable 9. Interrupted Time Series Estimates for Medicaid Insured Individuals After Dropping Low-Volume Facilities

This supplemental material has been provided by the authors to give readers additional information about their work.

© 2022 Steenland MW et al. *JAMA Network Open.*

eAppendix: Primary Regression Specification

The trends and level/trend changes in the quarterly percentage of deliveries with an associated immediate postpartum LARC in each state were estimated with the following regression specification:

$$Y_{ht} = \alpha + \beta * Time_{ht} + \delta * Post_{ht} + \gamma * TimePost_{ht} + \tau_h + \epsilon_{ht}$$

$Time_{ht}$ was a quarterly time trend being in the first quarter of 2011 through the last quarter of 2017. $Post_{ht}$ was an indicator equal to 1 for quarters after the start of the policy change and zero for quarters before the start of the policy change. $Time * Post_{ht}$ is an interaction term between the quarterly time trend and the post policy indicator. In the above equation, β captures the quarterly percentage point change in deliveries with IPP-LARC during the pre-policy period, δ captures the level change in the percentage of deliveries with IPP-LARC at the time of policy onset, γ captures the quarterly percentage point change in deliveries with IPP-LARC during the post-policy period relative to the pre-policy period, τ_h captures hospital fixed effects, and ϵ_{ht} is an error term, which allows for autocorrelation, heteroskedasticity, and cross-sectional dependence of observations within states. $TimePost_{ht}$ is defined such that it is 0 through the time of policy onset, after which it is incremented by 1.

© 2022 Steenland MW et al. *JAMA Network Open.*

eTable1: Diagnosis and Procedure Codes for Identification of Hospitalizations for Childbirth

Delivery Code(s)	Description
**ICD-9-CM**	
**V27**	Outcome of delivery (e.g., singleton, live; twins, one live one stillborn; other multiple births, all live)
**650**	Normal delivery
**72.0-72.9**	Selected procedures for forceps, vacuum, and breech deliveries
**73.22, 73.59, 73.6**	Other procedures inducing or assisting delivery
**74.0-74.99**	Cesarean section and removal of fetus
**ICD-10-CM**	
**Z37**	Outcome of delivery (e.g., single live birth; single stillbirth; quadruplets, some liveborn; unspecified)
**O80**	Encounter for delivery
**ICD-10-PCS**	
**10E0XZZ**	Spontaneous delivery
**10D00Z0-10D00Z2**	Cesarean delivery
**10D07Z3-10D07Z8**	Operative delivery
**DRG**	
**370-375**	Complicated cesarean section, uncomplicated cesarean section, complicated vaginal delivery, etc.
**765-775**	Cesarean delivery, vaginal delivery, etc.
**Exclusion Code(s)**	**Description**
**ICD-9-CM**	
**63.0-63.9**	Hydatidiform mole, abnormal production of conception, ectopic pregnancy, abortion, related complications
**69.01, 69.51**	Dilation/curettage for termination of pregnancy, aspiration curettage of uterus for termination of pregnancy
**74.91, 75.0**	Hysterotomy to terminate pregnancy, abortion of products of conception
**ICD-10-CM**	
**O00, O01, O02, O08**	Abnormal products of conception
**O03, O04, O07, Z33.2**	Abortion
**ICD-10-PCS**	
**10A07Z6, 10A07ZW, 10A07ZX, 10A07ZZ, 10A03ZZ, 10A04ZZ, 10A07ZX**	Abortion

© 2022 Steenland MW et al. *JAMA Network Open.*

eTable 2: Diagnosis and Procedure Codes for Identification of Immediate Postpartum LARC

Delivery Code(s)	Description
<u>**ICD-9 Diagnosis**</u>	
**V25.11, V25.5**	Encounter for insertion: intrauterine contraceptive, implantable subdermal contraceptive
<u>**ICD-9 Procedure**</u>	
**69.7**	Insertion of intrauterine contraceptive device
<u>**ICD-10 Diagnosis**</u>	
**Z30.014, Z30.017**	Encounter for initial prescription: intrauterine contraceptive, implantable subdermal contraceptive
**Z30.430**	Encounter for insertion of intrauterine contraceptive device
<u>**ICD-10 Procedure**</u>	
**0UH97HZ, 0UH98HZ**	Insertion of contraceptive device into uterus, via natural or artificial opening ± endoscopic
**0UHC7HZ,**	Insertion of contraceptive device into cervix, via natural or artificial opening ± endoscopic
**0UHC8HZ 0UH90HZ**	Insertion of contraceptive device into uterus, open approach
**0JH60HZ, 0JH63HZ**	Insertion of contraceptive device in chest subcutaneous tissue and fascia: open vs. perc
**0JH80HZ, 0JH83HZ**	Insertion of contraceptive device in abdominal subcutaneous tissue and fascia: open vs. perc
**0JHD0HZ, 0JHD3HZ**	Insertion of contraceptive device in right upper arm subcutaneous tissue and fascia: open vs. perc
**0JHF0HZ, 0JHF3HZ**	Insertion of contraceptive device in left upper arm subcutaneous tissue and fascia: open vs. perc
**0JHG0HZ, 0JHG3HZ**	Insertion of contraceptive device in right lower arm subcutaneous tissue and fascia: open vs. perc
**0JHH0HZ, 0JHH3HZ**	Insertion of contraceptive device in left lower arm subcutaneous tissue and fascia: open vs. perc
**0JHL0HZ, 0JHL3HZ**	Insertion of contraceptive device in right upper leg subcutaneous tissue and fascia: open vs. perc
**0JHM0HZ,**	Insertion of contraceptive device in left upper leg subcutaneous tissue and fascia: open vs. perc
**0JHM3HZ 0JHN0HZ,**	Insertion of contraceptive device in right lower leg subcutaneous tissue and fascia: open vs. perc
**0JHN3HZ 0JHP0HZ,**	Insertion of contraceptive device in left lower leg subcutaneous tissue and fascia: open vs. perc
**0JHP3HZ**	
<u>**CPT/HCPCS**</u>	
**11981**	Insertion, non-biodegradable drug delivery implant, Implanon or Nexplanon
**58300**	Insertion of IUD
**Q0090**	Levonorgestrel-releasing intrauterine contraceptive system, (Skyla), 13.5 mg
**J7297-J7307**	Contraceptive devices (e.g., levonorgestrel-releasing intrauterine/implant contraceptive system)
**S4981-S4989**	Insertion of levonorgestrel-releasing intrauterine system; intrauterine contraceptive, implant

© 2022 Steenland MW et al. *JAMA Network Open.*

eTable 3: Demographic Information by State of all Medicaid-Insured Individuals with Delivery Episodes Prior to Medicaid Reimbursement Policy Change

**Characteristic**	**% GA Deliveries n = 212 803**	**% IA Deliveries n = 40 364**	**% MD Deliveries n = 103 841**	**% NY Deliveries n = 350 347**	**% RI Deliveries n = 19 450**
Age					
12-17 years	4.2% (8,981 / 212,803)	3.2% (1,298 / 40,364)	3.4% (3,508 / 103,841)	2.4% (8,565 / 350,347)	3.2% (624 / 19,450)
18-29 years	71.8% (152,812 / 212,803)	76.5% (30,874 / 40,364)	67.0% (69,542 / 103,841)	63.6% (222,825 / 350,347)	68.6% (13,336 / 19,450)
30-35 years	17.6% (37,380 / 212,803)	15.7% (6,347 / 40,364)	21.5% (22,376 / 103,841)	23.7% (82,903 / 350,347)	20.6% (4,003 / 19,450)
36-50 years	6.4% (13,630 / 212,803)	4.6% (1,845 / 40,364)	8.1% (8,415 / 103,841)	10.3% (36,054 / 350,347)	7.6% (1,487 / 19,450)
Race/Ethnicity					
Hispanic	14.5% (30,726 / 211,261)	11.6% (4,374 / 37,588)	21.0% (21,007 / 99,897)	26.5% (92,565 / 349,287)	37.1% (6,626 / 17,872)
Non-Hispanic Black	45.8% (96,773 / 211,261)	11.5% (4,323 / 37,588)	44.0% (43,955 / 99,897)	20.7% (71,236 / 349,287)	12.2% (2,176 / 17,872)
Non-Hispanic White	33.2% (70,094 / 211,261)	71.8% (26,990 / 37,588)	26.3% (26,302 / 99,897)	27.5% (96,103 / 349,287)	45.6% (8,150 / 17,872)
Non-Hispanic Other	6.5% (13,668 / 211,261)	5.1% (1,901 / 37,588)	8.6% (8,633 / 99,897)	25.3% (88,483 / 349,287)	5.1% (920 / 17,872)
Below Median Education Zip Code[a]	76.4% (161,562 / 211,338)	43.7% (17,564 / 40,162)	---	81.2% (240,084 / 295,834)	72.5% (14,066 / 19,399)
Below 1st Quartile Income Zip Code	36.8% (77,617 / 211,128)	10.3% (4,136 / 40,127)	14.8% (15,291 / 103,239)	27.9% (82,326 / 295,501)	48.3% (9,353 / 19,380)
IPP-LARC Utilization[b]	0.2% (520 / 212,803)	0.3% (110 / 40,364)	0.6% (650 / 103,841)	0.2% (803 / 350,347)	0.9% (184 / 19,450)

Abbreviations: IPP-LARC, immediate postpartum long-acting reversible contraception

[a]Zip code level educational attainment data unavailable for MD
[b]IPP-LARC utilization refers to the share of deliveries accompanied by an inpatient LARC insertion in the immediate postpartum period.

© 2022 Steenland MW et al. *JAMA Network Open.*

eTable 4: Demographic Information for all Commercially Insured Individuals with Delivery Episodes Prior to Reimbursement Policy Change for Inpatient LARC[a]

**Characteristic**	**% Deliveries n = 751 219**
Age	
12-17 years	0.7% (5,141 / 751,219)
18-29 years	39.4% (295,845 / 751,219)
30-35 years	41.4% (311,213 / 751,219)
36-50 years	18.5% (139,020 / 751,219)
Race	
Hispanic	6.6% (48,290 / 736,454)
Non-Hispanic Black	13.7% (101,211 / 736,454)
Non-Hispanic White	66.1% (486,978 / 736,454)
Non-Hispanic Other	13.6% (99,975 / 736,454)
Below Median Education Zip Code[b]	44.4% (271,402 / 610,606)
Below 1st Quartile Income Zip Code	9.4% (70,431 / 746,128)
IPP-LARC Utilization[c]	0.0% (291 / 751,219)

Abbreviations: IPP-LARC, immediate postpartum long-acting reversible contraception

[a]Demographic information pooled across all states in the sample (GA, IA, MD, NY, RI).
[b]Zip code level educational attainment data unavailable for MD
[c]IPP-LARC utilization refers to the share of deliveries accompanied by an inpatient LARC insertion in the immediate postpartum period.

© 2022 Steenland MW et al. *JAMA Network Open.*

eTable 5: Testing for Structural Break Points in the Share of Medicaid-Insured Deliveries with Immediate Postpartum LARC Uptake

	Timing of Policy Change	Estimated Timing of Structural Breaks	F Statistic	P Value
**Georgia**	Q2 2014	Q4 2015	68.6	< 0.001
		Q3 2015	64.6	< 0.001
		Q2 2015	26.0	< 0.001
		Q1 2015	18.7	< 0.001
		**Q2 2014**	**18.4**	**< 0.001**
**Iowa**	Q1 2014	Q1 2016	16.9	< 0.001
		Q2 2016	9.5	< 0.001
		Q4 2015	9.0	0.001
		Q2 2014	4.2	0.027
		**Q1 2014**	**3.5**	**0.048**
**Maryland**	Q3 2014	Q3 2016	17.8	< 0.001
		Q2 2016	16.9	< 0.001
		Q4 2016	7.9	0.002
		Q1 2016	6.9	0.004
		Q3 2015	6.7	0.005
		Q4 2015	6.3	0.006
		Q2 2015	5.9	0.008
		Q4 2014	4.5	0.022
		Q1 2015	3.9	0.034
		**Q3 2014**	**3.3**	**0.055**
**New York**	Q2 2014	**Q2 2014**	**137.1**	**< 0.001**
**Rhode Island**	Q1 2015	Q3 2015	112.5	< 0.001
		Q4 2015	95.3	< 0.001
		Q4 2014	82.6	< 0.001
		**Q1 2015**	**82.5**	**< 0.001**

Abbreviations: IPP-LARC, immediate postpartum long-acting reversible contraception

Testing for the presence of structural breaks in time series of states' seasonality-adjusted share of deliveries with IPP-LARC uptake. Structural breaks are estimated using sequential Chow tests for break points between Q3 2011 and Q2 2017, with corresponding F statistics and p values. For each state, the results are reported in decreasing statistical significance until the estimated structural break aligns with the true timing of policy change. Unlike in Iowa and Maryland, the results for Georgia, New York, and Rhode Island suggest there is strong evidence of a structural break around the timing of policy change.

© 2022 Steenland MW et al. *JAMA Network Open.*

eTable 6: Adjusted Interrupted Time Series Estimates of Immediate Postpartum LARC Uptake Among Medicaid Insured Individuals Before and After Medicaid Reimbursement Change for LARC Provision

	**Georgia**[b]	**Iowa**[b]	**Maryland**[b]	**New York**[b]	**Rhode Island**[b]
Quarterly percentage point change during pre-policy period	-0.02<br>(-0.05, 0.02)<br>p = 0.39	0.07<br>(0.01, 0.14) *<br>p = 0.04	0.07<br>(0.05, 0.10) ***<br>p < 0.001	0.01<br>(0.00, 0.02)<br>p = 0.16	0.06<br>(0.01, 0.11) *<br>p = 0.03
Level change at time of policy onset	-0.17<br>(-0.54, 0.19)<br>p = 0.35	-0.28<br>(-0.63, 0.07)<br>p = 0.12	-0.30<br>(-0.62, 0.03)<br>p = 0.07	-0.02<br>(-0.13, 0.09)<br>p = 0.73	-0.16<br>(-1.20, 0.87)<br>p = 0.76
Quarterly percentage point change during post-policy period relative to pre-policy period	0.15<br>(0.12, 0.18) ***<br>p < 0.001	0.07<br>(0.01, 0.12) *<br>p = 0.02	0.00<br>(-0.03, 0.04)<br>p = 0.80	0.18<br>(0.17, 0.20) ***<br>p < 0.001	0.79<br>(0.69, 0.89) ***<br>p < 0.001
Overall change at end of Q4 2017 relative to estimated counterfactual[a]	1.93<br>(1.21, 2.66) ***<br>p < 0.001	0.65<br>(-0.20, 1.50)<br>p = 0.13	-0.24<br>(-0.80, 0.33)<br>p = 0.41	2.56<br>(2.32, 2.80) ***<br>p < 0.001	8.55<br>(7.53, 9.57) ***<br>p < 0.001

* $p < 0.05$ ** $p < 0.01$ *** $p < 0.001$

Abbreviations: IPP-LARC, immediate postpartum long-acting reversible contraception

[a]Absolute change in fraction of Medicaid insured deliveries with IPP-LARC placement. Estimated effects are relative to the predicted counterfactual fraction of deliveries with associated IPP-LARC through the end of Q4 2017 in all states.
[b]All estimates are adjusted for seasonality with quarter fixed effects, for clustering of observations by hospital with hospital fixed effects, and for state-quarter share of mothers of Black and/or Hispanic race/ethnicity, share of mothers from zip codes with below 25th percentile income, and share of mothers from zip codes with below median educational attainment. Driscoll-Kraay standard errors are used to account for autocorrelation, heteroskedasticity, and cross-sectional dependence of observations within states.

© 2022 Steenland MW et al. *JAMA Network Open.*

eTable 7: Interrupted Time Series Estimates for Iowa and New York Following Inclusion of Immediate Postpartum LARCs from State Ambulatory Data[a]

	**Iowa**[c]		**New York**[c]	
	Medicaid Insured	Commercially Insured	Medicaid Insured	Commercially Insured
Additional LARCs Observed in State Ambulatory Databases	5 (Among 145,304 Post-Policy Deliveries)		85 (Among 849,646 Post-Policy Deliveries)	
Quarterly percentage point change during pre-policy period	0.08 (0.05, 0.11) *** p < 0.001	0.02 (0.01, 0.03) *** p < 0.001	0.00 (0.00, 0.01) p = 0.22	0.00 (0.00, 0.00) ** p = 0.01
Level change at time of policy onset	-0.28 (-0.62, 0.07) p = 0.11	0.04 (-0.08, 0.15) p = 0.55	-0.07 (-0.22, 0.08) p = 0.35	0.01 (-0.01, 0.03) p = 0.38
Quarterly percentage point change during post-policy period relative to pre-policy period	0.05 (0.00, 0.11) * p = 0.05	0.00 (-0.02, 0.01) p = 0.72	0.17 (0.15, 0.19) *** p < 0.001	0.03 (0.02, 0.03) *** p < 0.001
Overall change at end of Q4 2017 relative to estimated counterfactual[b]	0.48 (-0.38, 1.34) p = 0.28	-0.01 (-0.29, 0.28) p = 0.97	2.32 (2.13, 2.52) *** p < 0.001	0.38 (0.35, 0.42) *** p < 0.001

* $p < 0.05$ ** $p < 0.01$ *** $p < 0.001$

Abbreviations: IPP-LARC, immediate postpartum long-acting reversible contraception

[a]Given Medicaid program guidelines in Iowa and New York to bill IPP-LARCs on separate ambulatory claims, we supplemented inpatient data with State Ambulatory Surgery and Services Databases from HCUP in these two states.
[b]Absolute change in fraction of Medicaid insured deliveries with IPP-LARC placement. Estimated effects are relative to the predicted counterfactual fraction of deliveries with associated IPP-LARC through the end of Q4 2017 in all states.
[c]All estimates are adjusted for seasonality with quarter fixed effects and for clustering of observations by hospital with hospital fixed effects. Driscoll and Kraay standard errors are used to account for autocorrelation, heteroskedasticity, and cross-sectional dependence of observations within states.

© 2022 Steenland MW et al. *JAMA Network Open.*

Interrupted Time Series Estimates for Medicaid Insured Individuals in Georgia, Iowa, New York, and Rhode Island with Deliveries
eTable 8: Aggregated at the Monthly Level[a]

	**Georgia**[c]	**Iowa**[c]	**New York**[c]	**Rhode Island**[c]
Monthly percentage point change during pre-policy period	0.00 (-0.01, 0.00) p = 0.20	0.03 (0.02, 0.04) *** p < 0.001	0.00 (0.00, 0.00) p = 0.15	0.01 (-0.01, 0.03) p = 0.22
Level change at time of policy onset	-0.19 (-0.46, 0.09) p = 0.18	-0.34 (-0.73, 0.05) p = 0.08	-0.11 (-0.23, 0.00) p = 0.06	-0.13 (-1.18, 0.91) p = 0.80
Monthly percentage point change during post-policy period relative to pre-policy period	0.05 (0.04, 0.06) *** p < 0.001	0.02 (0.00, 0.03) * p = 0.04	0.06 (0.05, 0.06) *** p < 0.001	0.27 (0.24, 0.31) *** p < 0.001
Overall change at end of Dec 2017 relative to estimated counterfactual[b]	1.94 (1.45, 2.43) *** p < 0.001	0.42 (-0.50, 1.34) p = 0.37	2.32 (2.17, 2.47) *** p < 0.001	9.43 (8.28, 10.59) *** p < 0.001

* $p < 0.05$ ** $p < 0.01$ *** $p < 0.001$

Abbreviations: IPP-LARC, immediate postpartum long-acting reversible contraception

[a]Maryland not included, as only discharge quarter (no admission or discharge month) provided.
[b]Absolute change in fraction of Medicaid insured deliveries with IPP-LARC placement. Estimated effects are relative to the predicted counterfactual fraction of deliveries with associated IPP-LARC through the end of 2017 in all states.
[c]All estimates are adjusted for seasonality with month fixed effects and for clustering of observations by hospital with hospital fixed effects. Driscoll and Kraay standard errors are used to account for autocorrelation, heteroskedasticity, and cross-sectional dependence of observations within states.

© 2022 Steenland MW et al. *JAMA Network Open.*

eTable 9: Interrupted Time Series Estimates for Medicaid Insured Individuals After Dropping Low-Volume Facilities[a]

	**Georgia**[c]	**Iowa**[c]	**Maryland**[c]	**New York**[c]	**Rhode Island**[c]
Facilities Dropped	50/95	75/81	21/45	95/164	8/9
Quarterly percentage point change during pre-policy period	-0.01<br>(-0.03, 0.01)<br>p = 0.19	0.18<br>(0.10, 0.25) ***<br>p < 0.001	0.07<br>(0.05, 0.08) ***<br>p < 0.001	0.00<br>(0.00, 0.01)<br>p = 0.31	0.03<br>(-0.01, 0.07)<br>p = 0.10
Level change at time of policy onset	-0.18<br>(-0.55, 0.18)<br>p = 0.33	-0.58<br>(-1.43, 0.26)<br>p = 0.18	-0.26<br>(-0.71, 0.18)<br>p = 0.24	-0.04<br>(-0.17, 0.09)<br>p = 0.54	-0.24<br>(-1.32, 0.83)<br>p = 0.64
Quarterly percentage point change during post-policy period relative to pre-policy period	0.16<br>(0.12, 0.20) ***<br>p < 0.001	0.11<br>(-0.02, 0.24)<br>p = 0.09	0.04<br>(-0.01, 0.09)<br>p = 0.09	0.18<br>(0.17, 0.20) ***<br>p < 0.001	1.03<br>(0.90, 1.16) ***<br>p < 0.001
Overall change at end of Q4 2017 relative to estimated counterfactual[b]	2.04<br>(1.47, 2.61) ***<br>p < 0.001	0.96<br>(-1.12, 3.03)<br>p = 0.37	0.22<br>(-0.13, 0.57)<br>p = 0.21	2.53<br>(2.32, 2.75) ***<br>p < 0.001	11.09<br>(10.20, 11.97) ***<br>p < 0.001

* $p < 0.05$ ** $p < 0.01$ *** $p < 0.001$

Abbreviations: IPP-LARC, immediate postpartum long-acting reversible contraception

[a]Low-volume hospitals are defined as in Handley et al. (2021), namely hospitals with an average number of deliveries per year less than 500.

[b]Absolute change in fraction of Medicaid insured deliveries with IPP-LARC placement. Estimated effects are relative to the predicted counterfactual fraction of deliveries with associated IPP-LARC through the end of Q4 2017 in all states.

[c]All estimates are adjusted for seasonality with quarter fixed effects and for clustering of observations by hospital with hospital fixed effects. Driscoll and Kraay standard errors are used to account for autocorrelation, heteroskedasticity, and cross-sectional dependence of observations within states.

© 2022 Steenland MW et al. *JAMA Network Open.*
